# Antibiotic-sparing strategies for multidrug-resistant organism (MDRO) infections

**DOI:** 10.3389/fphar.2025.1653424

**Published:** 2025-09-29

**Authors:** Shuai Geng, Qing Tang, Ning Shi

**Affiliations:** Department of Pharmacy, The Ninth Medical Center of PLA General Hospital, Beijing, China

**Keywords:** antibiotic-sparing, MDROs, non-antibiotic therapies, antimicrobial stewardship, infection

## Abstract

The global rise of multidrug-resistant organisms (MDROs), such as *carbapenem-resistant Enterobacteriaceae* (CRE) and *methicillin-resistant Staphylococcus* aureus (MRSA), has rendered conventional antibiotics increasingly ineffective, particularly in intensive care units (ICUs) where mortality rates exceed 50% in severe infections. Overuse of broad-spectrum antibiotics accelerates resistance while disrupting host microbiota, necessitating innovative “antibiotic-sparing” strategies. This review synthesizes three pillars of intervention: (1) non-antibiotic therapies, including *bacteriophages* for targeted pathogen lysis, monoclonal antibodies (e.g., BiS4αPa against *Pseudomonas aeruginosa*), and nanotechnology-enhanced *antimicrobial peptides* (AMPs) for biofilm disruption; (2) antimicrobial stewardship integrating rapid diagnostics (MALDI-TOF, mNGS), PK/PD-guided dosing, and short-course regimens (7-day therapy validated by RCTs); and (3) transmission prevention through UV-C disinfection, AI-driven hygiene compliance, and gut microbiota modulation. Key innovations include phage-antibiotic synergies, bispecific antibody engineering, and dynamic PK/PD-TDM frameworks. Despite challenges in clinical translation and cost-effectiveness, these strategies collectively reduce antibiotic reliance, mitigate resistance evolution, and offer a paradigm shift toward precision infection control. Future directions emphasize combinatorial therapies, regulatory harmonization, and scalable environmental-behavioral interventions to address the post-antibiotic era crisis.

## 1 Introduction

The global spread of multidrug-resistant organisms (MDROs), including *carbapenem-resistant Enterobacteriaceae* (CRE), *methicillin-resistant Staphylococcus aureus* (MRSA), and *Acinetobacter baumannii*, has escalated into a critical public health crisis. The World Health Organization (WHO) explicitly lists these pathogens as priority targets in its “Priority Pathogens” list for antimicrobial resistance, emphasizing their high mortality rates and limited therapeutic options (GBD, 2021 Antimicrobial Resistance Collaborators, 2024; Tacconelli et al., 2018). In intensive care units (ICUs), where immunocompromised patients and invasive devices converge, MDRO infections account for over 40% of healthcare-associated infections, with mortality rates exceeding 50% in carbapenem-resistant *Klebsiella pneumoniae* (CRKP) bloodstream infections (Tabah et al., 2020).

The overuse and misuse of antibiotics remain the primary drivers of resistance development. Between 2000 and 2015, global antibiotic consumption surged by 65%, with up to 70% of ICU patients receiving empirical broad-spectrum antibiotics (Pulingam et al., 2022). This practice not only accelerates resistance but also disrupts host microbiota, increasing risks of Clostridioides difficile infections and secondary fungal colonization (Isles et al., 2022). Conventional antibiotic therapy faces growing limitations: even “last-resort” agents like polymyxins exhibit rising resistance rates (e.g., 30% polymyxin resistance in CRKP), while prolonged courses induce nephrotoxicity and ecological collateral damage (Silva et al., 2022).

To address these challenges, the concept of “antibiotic de-escalation” has evolved from its original definition—narrowing antibiotic spectra or shortening duration based on clinical stability—to a broader “de-antibiotization” strategy (Vallicelli et al., 2022). This framework integrates three pillars: (1) reducing antibiotic exposure through stewardship programs; (2) employing non-antibiotic therapies to directly target MDROs; and (3) interrupting transmission via environmental and microbiome interventions (Campion and Scully, 2018; Lanckohr and Bracht, 2022). For instance, phage therapy has successfully rescued patients with CRKP intracranial abscesses unresponsive to carbapenems (Zhao et al., 2024), while monoclonal antibodies targeting *Pseudomonas aeruginosa* exotoxins have reduced mortality in ventilator-associated pneumonia (Simon et al., 2023). Plant extracts, notably essential oils, demonstrate significant therapeutic potential against oral pathogens by effectively inhibiting biofilm formation and reducing their pathogenicity (Radu et al., 2023). Their bioactive components exert antimicrobial effects primarily by disrupting microbial cell membranes. Furthermore, incorporating these plant-derived compounds into nanoparticles can enhance their stability and bioavailability, enabling more efficient targeted antimicrobial delivery. Nanoparticles themselves can also exert synergistic antibacterial effects through physical disruption or by generating reactive oxygen species (Radu et al., 2023).

This review synthesizes recent advances and challenges in “de-antibiotization” strategies for ICU-acquired MDRO infections, critically evaluates the efficacy of novel therapies, stewardship pathways, and preventive measures, and discusses barriers to clinical translation and policy adaptation.

## 2 Non-antibiotic therapies

### 2.1 Phage therapy

*Bacteriophages (phages)* are naturally occurring viruses that exert therapeutic effects by specifically recognizing and infecting bacterial hosts. Their core mechanisms encompass three aspects: ① Lytic Cycle: Lytic phages adsorb to bacterial surface receptors (e.g., outer membrane proteins or flagella), inject genetic material into the host cell, hijack its metabolic machinery for rapid replication, and ultimately lyse the cell to release progeny phages. This process directly eliminates pathogens and amplifies bactericidal effects through cascade infections (Strathdee et al., 2023). ② Precision Targeting: Phages exhibit exceptional host specificity, infecting only particular bacterial species or strains, thereby sparing commensal microbiota (Uyttebroek et al., 2022). ③ Anti-Biofilm Activity: Phage-derived lysins degrade biofilm matrices, penetrate bacterial aggregates on medical devices (e.g., prosthetic valves or catheters), and enhance antibiotic permeability (Uyttebroek et al., 2022).

With the rising incidence of multidrug-resistant (MDR) infections, the clinical value of *bacteriophage* therapy (BT) as an antibiotic alternative has prompted extensive exploration. Research by Graham F. Hatfull’s team highlights the profound global public health threat posed by bacterial resistance (Hatfull et al., 2022), a context that drove the establishment of the Center for Innovative Phage Applications and Therapeutics (IPATH) at the University of California, San Diego, in June 2018 to systematically advance BT clinical translation. A retrospective analysis by Saima Aslam’s team of 785 BT consultation requests at IPATH within its first 2 years revealed that *Pseudomonas aeruginosa*, *Staphylococcus aureus*, and *mycobacterial* infections accounted for one-third of inquiries, with 17 patients ultimately receiving BT (10 via intravenous administration). Notably, while 7 patients achieved infection clearance (including 6 treated pre-IPATH), 2 cases showed limited efficacy due to failed *in vitro* phage susceptibility testing, underscoring the need for optimized phage screening strategies (Aslam et al., 2020). Studies confirm the safety of intravenous BT, with outpatient self-administration models and synergistic antibiotic combinations offering novel avenues to overcome resistance mechanisms. However, phage resistance management still requires dynamic adjustments to phage cocktails.

In specific clinical scenarios, Evgenii Rubalskii’s team investigated BT in critical post-cardiot thoracic surgical infections: 8 immunocompromised patients (aged 13–66 years) with vascular graft/device infections or post-transplant complications received personalized BT (local/oral/inhaled) after conventional antibiotic failure. After a mean 48.5 ± 16.7 days of bacteriological monitoring, 7 patients (87.5%) achieved target pathogen eradication without severe adverse events, validating BT as a safe salvage therapy for MDR infections in cardiothoracic surgery (Rubalskii et al., 2020). These findings align with Hatfull’s emphasis on BT’s strategic value—prolonging existing antibiotics’ lifespan through synergy and buying critical time for novel antibiotic development (Hatfull et al., 2022). Nevertheless, challenges persist, including strain heterogeneity-induced targeting limitations, host immune interference, and phage resistance evolution. These demand improved cocktail design, precision delivery systems, and robust resistance surveillance to establish multidimensional solutions for post-antibiotic era infection control (Hatfull et al., 2022; Aslam et al., 2020; Rubalskii et al., 2020).

### 2.2 Monoclonal antibody

In the treatment of multidrug-resistant organism (MDRO) infections, “antibiotic-sparing” strategies reduce reliance on conventional antibiotics by targeting bacterial pathogenic mechanisms and enhancing host immune defenses, offering innovative solutions to the resistance crisis. *Monoclonal antibody* (mAb) therapy represents a pivotal direction in this field.

*Monoclonal antibodies* exert therapeutic effects by specifically binding critical bacterial virulence factors, impairing infectivity and promoting immune clearance, thereby avoiding resistance risks associated with direct bactericidal pressure. A representative application involves antibodies targeting *Klebsiella pneumoniae* capsular polysaccharides (Alfaleh et al., 2020). This polysaccharide inhibits complement activation and phagocyte recognition, serving as a core virulence factor for immune evasion. Specific mAbs neutralize the polysaccharide’s immune-shielding effects, enhancing opsonophagocytosis by neutrophils and macrophages, which significantly reduces bacterial loads in pulmonary or bloodstream infections (Buss et al., 2012). Similar strategies can be extended to other MDROs, such as antibodies targeting biofilm-forming proteins in *Acinetobacter* baumannii. These mAbs selectively neutralize virulence factors with minimal disruption to host microbiota, while engineering modifications (e.g., bispecific antibodies) can prolong half-life or enhance neutralizing potency (Stone et al., 2024).

Antonio DiGiandomenico’s team addressed *Pseudomonas aeruginosa* resistance by developing BiS4αPa, a bispecific antibody targeting the virulence factor PcrV protein and biofilm-associated Psl polysaccharide (DiGiandomenico et al., 2014). This antibody operates through triple synergistic mechanisms: blocking bacterial type III secretion system-mediated cytotoxicity, neutralizing exopolysaccharides to inhibit biofilm formation, and enhancing immune phagocytosis via its Fc region. In murine pneumonia models, BiS4αPa demonstrated broad-spectrum anti-infective activity and synergistic effects with multiple antibiotics, significantly improving bactericidal efficiency. Its clinical candidate MEDI3902 validates the potential of “multi-mechanism synergy,” establishing a novel “precision attenuation–immune synergy” paradigm for resistant infections, adaptable to other MDROs like A. baumannii.

Bruno François’ team conducted a multicenter randomized double-blind phase II trial (NCT02296320) to evaluate the α-toxin mAb suvratoxumab for preventing ventilator-associated pneumonia (VAP) caused by *Staphylococcus aureus* in ICU patients (François et al., 2021). The study enrolled 213 critically ill patients with lower respiratory tract S.aureus colonization from 31 European hospitals, randomized to receive suvratoxumab 5000 mg or placebo. Results showed a 30-day VAP incidence of 18% (17/96) in the 5000 mg group versus 26% (26/100) in the placebo group, indicating a 31.9% relative risk reduction trend (90% CI: −7.5–56.8), though statistical significance was not achieved. Treatment-related adverse events (91% vs. 92%) and serious adverse events (38% vs. 32%) were comparable between groups. Despite not meeting the primary endpoint, suvratoxumab demonstrated potential VAP risk reduction with favorable safety, suggesting virulence-targeting mAbs may reduce antibiotic dependence. However, dose optimization or combination strategies require validation in phase III trials.

### 2.3 Antimicrobial peptides & nanotechnology

*Antimicrobial peptides* (AMPs) are short-chain amino acid-based molecules with broad-spectrum antimicrobial activity, serving as essential components of the innate immune systems in mammals, fungi, and plants. Unlike conventional antibiotics, AMPs exhibit unique bactericidal mechanisms, including disrupting membrane integrity (via pore formation), inhibiting cell wall synthesis, interfering with intracellular processes, and suppressing quorum sensing (Luo and Song, 2021). For instance, LL-37, a human-derived AMP, interacts with negatively charged phospholipids on bacterial membranes to form transmembrane pores, leading to cytoplasmic leakage and bacterial death (Wei et al., 2022). However, clinical applications of AMPs are often limited by protease degradation and poor biofilm penetration. To address these challenges, nanotechnology has been employed to optimize their performance: nanoparticle-based delivery systems not only enhance AMP stability (e.g., bacterial-responsive self-assembling AMPs constructed via hydrogen bonding modules to mitigate trypsin susceptibility) but also improve bioavailability, enable targeted delivery to elevate local drug concentrations, and augment biofilm penetration (Mut and eeb, 2023). Additionally, nanomaterials themselves possess inherent antimicrobial potential. Silver nanoparticles (AgNPs), for example, exhibit broad-spectrum antibacterial activity with low resistance induction. Studies demonstrate that AgNPs at sub-inhibitory concentrations significantly inhibit biofilm formation in *Pseudomonas aeruginosa*. AgNPs synthesized using Isodon rugosus extract achieved 78% inhibition of *P. aeruginosa* biofilm formation at sub-MIC concentrations while disrupting mature biofilms (Bruna et al., 2021). Furthermore, nanoparticle systems synergize with antibiotics. AgNPs combined with antibiotics markedly enhance bactericidal efficacy against biofilm-embedded bacteria. For example, in photodynamic therapy (PDT), AgNPs conjugated with ciprofloxacin (CIP) achieved 99.99% *in vitro* antibacterial efficiency against *methicillin-resistant Staphylococcus* aureus (MRSA) biofilms (Morones-Ramirez et al., 2013).

The clinical translation of AMPs and nanotechnology is progressing. Research by Jessica’s team highlights that diabetic foot ulcers (DFUs), affecting approximately 15% of diabetic patients, represent the leading cause of non-traumatic amputations, with polymicrobial bacterial-fungal infections exacerbating tissue necrosis and worsening prognosis (Da Silva et al., 2021). Although current literature inadequately addresses the risks of polymicrobial infections, AMPs emerge as ideal candidates for DFU treatment due to their broad-spectrum activity (encompassing bacteria, fungi, and viruses) and wound-healing properties. AMPs selectively target aerobic/anaerobic bacteria and fungi, while their molecular mechanisms (e.g., membrane disruption and immunomodulation) confer advantages over traditional antibiotics. Nevertheless, AMPs’ antifungal mechanisms and synergistic roles in wound repair require further elucidation. Current studies emphasize that integrating nanotechnology (e.g., targeted delivery systems) can unlock AMPs’ clinical potential by enhancing stability, bioavailability, and dual antimicrobial-pro-healing effects, offering novel therapeutic strategies for chronic polymicrobial wounds like DFUs. Such integrative approaches not only address antimicrobial resistance but also open new avenues for translational research.

## 3 Antibiotics management

In the complex clinical setting of the intensive care unit (ICU), the specialized antimicrobial stewardship (AMS) pathway requires multidimensional intervention strategies to balance precision anti-infective therapy with antimicrobial resistance control. Given the characteristic pathophysiological disturbances in ICU patients and the high prevalence of multidrug-resistant pathogens, de-escalation therapy and short-course strategies form the core framework of dynamic antimicrobial regimens. Following early broad-spectrum antibiotic administration (“hit hard and early”) to control sepsis progression, the integration of rapid diagnostic technologies (RDTs) – including multiplex PCR, matrix-assisted laser desorption ionization time-of-flight mass spectrometry (MALDI-TOF), and metagenomic next-generation sequencing (mNGS) – enables timely antibiotic spectrum narrowing and treatment duration reduction to 5–7 days based on pathogen identification. This approach significantly mitigates selective pressure and secondary infection risks, while bedside dynamic monitoring of inflammatory biomarkers (e.g., procalcitonin-guided discontinuation) facilitates individualized treatment duration optimization.

For the prevalent pharmacokinetic/pharmacodynamic (PK/PD) parameter alterations in critically ill patients, a dual-modality dosing strategy combining population pharmacokinetic model-based feed-forward dosing optimization with therapeutic drug monitoring (TDM)-guided feedback adjustments proves essential. Real-time blood concentration tracking of high-variability agents such as vancomycin and β-lactams transcends traditional fixed-dose paradigms, enabling restoration of therapeutic exposure thresholds under conditions of organ dysfunction or extracorporeal life support. This closed-loop management pathway–characterized by “rapid diagnostics-driven de-escalation” coupled with “dynamic PK/PD-anchored precision dosing” – achieves synergistic effects through temporally coordinated multidimensional interventions. Ultimately, it drives the paradigm shift from empirical broad-spectrum coverage to targeted antimicrobial therapy in critical care medicine.

### 3.1 De-escalation & short-course therapy

Under the antimicrobial stewardship (AMS) framework, de-escalation therapy and short-course strategies are pivotal approaches to achieving precision anti-infective therapy and mitigating antimicrobial resistance risks. De-escalation emphasizes narrowing the antibiotic spectrum to targeted agents after pathogen identification, thereby reducing selective pressure, while short-course strategies minimize unnecessary antibiotic exposure by truncating treatment duration. Recent large-scale randomized controlled trials (RCTs) have provided robust evidence supporting these approaches. For instance, the BALANCE trial (NCT03005145) involving 3,608 patients with bloodstream infections demonstrated that a 7-day antibiotic course achieved non-inferior 90-day mortality (14.5%) compared to a 14-day regimen (16.1%), with significantly reduced antibiotic exposure (median 8 vs. 14 days) and no increased risk of relapse or secondary resistant infections, supporting the feasibility of short-course therapy in critically ill patients (Daneman et al., 2025). Another RCT focused on Gram-negative bacteremia (NCT01737320) further revealed comparable composite endpoints (mortality, relapse, and readmission rates) between 7-day and 14-day courses in clinically stabilized patients (45.8% vs. 48.3%), with accelerated functional recovery observed in the short-course group (Yahav et al., 2019). Similarly, the SHORTEN trial (NCT02400268) for Enterobacteriaceae bloodstream infections, utilizing Desirability of Outcome Ranking/Response Adjusted for Duration of Antibiotic Risk (DOOR/RADAR) analysis, demonstrated equivalent clinical cure rates (93.6% vs. 89.9%) between 7-day and 14-day regimens, alongside reduced antibiotic exposure (median 7 vs. 14 days) and superior overall benefit-risk profiles (77.7% probability favoring 7-day therapy) (Molina et al., 2022). Collectively, these studies indicate that rapid diagnostic-driven de-escalation (e.g., MALDI-TOF, mNGS) coupled with short-course interventions synergistically optimizes antimicrobial use and reduces selection pressure for resistant pathogens. Furthermore, analogous principles have been extended to pneumonia and intra-abdominal infections, exemplified by non-inferior outcomes of 7-day versus 10-day courses in community-acquired pneumonia (Uranga et al., 2016) and emerging evidence supporting short-course therapy for complicated intra-abdominal infections (Sawyer et al., 2015), reinforcing the generalizability of AMS strategies. In summary, dynamic antibiotic spectrum adjustment and duration optimization under AMS frameworks maintain clinical efficacy while significantly curbing cumulative resistance risks, driving the paradigm shift from empirical broad-spectrum coverage to precision-targeted therapy.

### 3.2 Rapid diagnostic technologies

The application of rapid diagnostic technologies in antimicrobial stewardship has significantly optimized antibiotic utilization strategies, effectively reducing resistance risks and promoting antibiotic de-escalation. By rapidly identifying pathogens and their resistance genes, these technologies enable clinicians to promptly adjust treatment regimens, thereby minimizing the overuse of empirical broad-spectrum antibiotics. For example, MALDI-TOF MS technology achieves pathogen identification within hours through analysis of microbial-specific protein profiles. When integrated with antimicrobial stewardship programs (ASPs), its application reduces the time to effective therapy by an average of 19.7 h, shortens hospital stays by 2.8 days, and significantly lowers 30-day mortality rates (Briggs et al., 2021; Beganovic et al., 2017). Molecular diagnostic platforms such as BioFire FilmArray™ and Accelerate Pheno™ further incorporate phenotypic susceptibility testing, enabling antibiotic optimization within 7 h and reducing broad-spectrum antibiotic exposure duration (Briggs et al., 2021). Meta-analysis has demonstrated that molecular rapid diagnostic tests (mRDT) combined with antimicrobial stewardship programs (ASP) can reduce the mortality rate of patients with bloodstream infections by 36% and shorten the length of hospital stay by 2.48 days, with particularly significant effects observed in infections caused by multidrug-resistant Gram-negative bacteria (Timbrook et al., 2017). Taking the Verigene^®^ system as an example, its ability to perform real-time detection of resistance genes (such as *mecA* and *vanA*), when combined with ASP interventions, can advance the time to antibiotic optimization by 15.7 h and reduce hospitalization costs by $11,661 (Rivard et al., 2017).

Furthermore, the study by Perez et al. revealed (Perez et al., 2014) that the direct application of MALDI-TOF MS combined with ASP for the management of bloodstream infections caused by multidrug-resistant Gram-negative bacteria shortened the treatment duration by 57.7 h, reduced hospitalization costs by $26,298, and decreased the mortality rate by 12.1%. The successful implementation of a de-escalation strategy is also evident in the management of coagulase-negative staphylococci (CoNS) bloodstream infections. By rapidly differentiating between contamination and true infection, MALDI-TOF MS technology guided by ASP reduced unnecessary antibiotic courses by 2.58 days (Nagel et al., 2014). These cases underscore the necessity of the synergistic effect between rapid diagnostic techniques and ASP: without real-time feedback and clinical guidance, even if the technology itself shortens the detection time, it is difficult to translate this into clinical benefits. Therefore, future research should further explore how to achieve more precise antibiotic de-escalation therapy through the integration of standardized ASP processes with emerging diagnostic technologies (such as metagenomic sequencing), thereby curbing the global spread of antibiotic-resistant bacteria.

### 3.3 PK/PD & TDM

In antimicrobial stewardship, pharmacokinetic/pharmacodynamic (PK/PD)-based dose optimization combined with therapeutic drug monitoring (TDM) serves as a critical strategy for reducing resistance development and facilitating antibiotic de-escalation. In critically ill patients, pathophysiological alterations often induce significant fluctuations in antibiotic exposure, where standard dosing regimens may fail to achieve therapeutic concentrations or induce toxicity.

A meta-analysis of beta-lactam antibiotics demonstrated that TDM-guided dose adjustments significantly improved target attainment rates (RR 1.85), clinical cure (RR 1.17), and microbiological eradication (RR 1.14), without increasing mortality, highlighting TDM’s value in optimizing efficacy and reducing treatment failure (Pai Mangalore et al., 2022). The REGARD-VAP trial in ventilator-associated pneumonia (VAP) further revealed that personalized short-course antibiotic therapy (median 6 days) based on clinical response (e.g., fever resolution and hemodynamic stability) showed no significant difference in 60-day mortality or recurrence compared to conventional prolonged courses (median 14 days) (risk difference of −3%), but markedly reduced antibiotic-associated adverse events (38% vs. 8%) and acute kidney injury incidence (35% vs. 5%) (Mo et al., 2024). These findings support de-escalation through shortened duration and dynamic clinical monitoring to mitigate resistance selection pressure. Other studies validate similar strategies: the ProACT trial demonstrated that procalcitonin-guided antibiotic duration reduction shortened treatment from 13 to 11 days (Schuetz et al., 2017), while the SAPS-TDM study showed TDM-guided vancomycin dosing significantly lowered nephrotoxicity risk (OR 0.36) (Neely et al., 2018). Additionally, TDM application for aminoglycosides optimizes peak concentration-to-MIC ratios, reducing ototoxicity/nephrotoxicity while enhancing efficacy (Roberts et al., 2014). Collectively, the integration of PK/PD optimization with TDM not only improves therapeutic outcomes in critically ill patients but also reduces antibiotic exposure through precision dosing and shortened courses, providing evidence-based support for antimicrobial stewardship and resistance containment.

## 4 Transmission prevention

The containment of multidrug-resistant organism (MDRO) transmission is enabling a “non-antibiotic-dependent” prevention paradigm through innovations in environmental disinfection technologies, gut microbiota modulation, and multimodal infection control strategies. Addressing traditional disinfection blind spots, non-contact dry mist hydrogen peroxide technology achieves a 6 log_10_ reduction (≥99.9999% kill rate) against *Acinetobacter* baumannii and carbapenem-resistant Enterobacterales (CRE) via micron-scale particle penetration, while minimizing metal instrument corrosion risks, thereby ensuring terminal disinfection in high-risk areas (Russotto et al., 2017). At the gut microbiota level, probiotic interventions (e.g., *Lactobacillus* rhamnosus GG) reduce gastrointestinal colonization density of opportunistic pathogens (e.g., *Pseudomonas aeruginosa*) by 30%–50%, and combined with metagenomic resistance gene profiling, enable precise identification of high-risk colonized individuals to guide targeted decolonization (Forslund et al., 2013). In infection control, multimodal strategies integrating active surveillance, contact isolation, fluorescent marker-guided cleaning validation (compliance rate increased to 92%), real-time PCR environmental surveillance (sensitivity ≥95%), and whole-genome sequencing (WGS)-based transmission chain analysis reduce nosocomial outbreak risks by over 40% (Simões et al., 2022). This comprehensive environment-host-behavioral prevention model is progressively replacing antibiotic-reliant passive defenses, offering sustainable solutions for MDRO transmission control.

### 4.1 Environmental disinfection

The recent application of novel environmental disinfection technologies has significantly improved the clearance of multidrug-resistant organism (MDRO) colonization in high-risk areas such as ICUs. Ultraviolet-C (UV-C) robots, as representative non-contact disinfection systems, have demonstrated high efficacy in multiple studies. Research by Casini et al. revealed that UV-C combined with standard cleaning protocols reduced microbial contamination positivity rates on high-touch surfaces in ICUs and operating rooms from 64.3% to 17.5%, with non-compliant sampling points decreasing from 9.3% to 1.2%. This approach exhibited particularly notable efficacy against Gram-negative bacilli (e.g., *Pseudomonas aeruginosa*, *Acinetobacter* baumannii) and *methicillin-resistant Staphylococcus aureus* (MRSA) (Casini et al., 2018). A separate controlled trial focusing on Gram-negative bacteria demonstrated that UV-C irradiation achieved an average 2.35 log reduction in colony-forming units (CFU), with effectiveness against both non-fermenters (e.g., A. baumannii) and Enterobacteriaceae (e.g., *Klebsiella pneumoniae*), though disinfection blind spots persisted in distant or shielded areas (Anderson et al., 2018). While hydrogen peroxide nebulization systems were not directly evaluated in these studies, their synergistic use with UV-C is frequently proposed to enhance biofilm disruption and disinfection coverage through oxidative mechanisms. Although antimicrobial coating materials (e.g., copper alloy surfaces) remain undetailed in current literature, prior studies suggest their capacity to inhibit bacterial survival via continuous metal ion release, thereby reducing environmental colonization (Rudhart et al., 2022). Collectively, UV-C-centered multimodal environmental interventions, when integrated with standardized cleaning protocols and material innovations, substantially mitigate MDRO transmission risks, providing critical support for “antibiotic de-escalation” strategies.

### 4.2 Intestinal flora protection and selective decolonization

In the “de-antibiotic” strategy for managing infections caused by multidrug-resistant organisms (MDROs), the core of blocking the transmission of resistant bacteria lies in balancing infection control with the protection of the gut microbiota. Selective Digestive Decontamination (SDD), as an intervention measure, reduces the colonization of potential pathogens in the oropharynx and gastrointestinal tract through the local application of non-absorbable antibiotics (such as polymyxins, aminoglycosides, and antifungal agents), thereby lowering the risk of ventilator-associated pneumonia (VAP) and bloodstream infections. In mechanically ventilated patients in the ICU, the application of SDD has been confirmed by multiple studies to significantly reduce hospital mortality, especially when combined with intravenous antibiotics (Hammond et al., 2022). However, the widespread promotion of SDD remains controversial: on the one hand, it may disrupt the diversity of the gut microbiota, increasing the selective pressure on resistant bacteria (such as *methicillin-resistant Staphylococcus aureus* and vancomycin-resistant *Enterococcus*); on the other hand, existing evidence suggests that the long-term impact of SDD on the colonization of resistant bacteria is still unclear, and there is heterogeneity in the assessment of resistance risks across different studies (Tsuchiya et al., 2024). For instance, some studies have observed no significant changes in the detection rates of resistant bacteria after SDD use, while others have suggested that resistance rates may rise in specific environments, particularly when antibiotic stewardship protocols are not strictly followed (Hammond et al., 2022).

To reduce reliance on traditional antibiotics, strategies for protecting the gut microbiota have gradually gained attention. Probiotics (such as *Saccharomyces* boulardii) and prebiotics can reduce the risk of intestinal colonization by MDROs through competitive inhibition of pathogen colonization, enhancement of intestinal barrier function, and modulation of immune responses. Studies have shown that S. boulardii can effectively reduce antibiotic-associated diarrhea and *Clostridium difficile* infections, but its role in preventing the colonization of multidrug-resistant Gram-negative bacteria still requires more clinical validation (Tsuchiya et al., 2024). In addition, targeted decolonization against specific pathogens (such as the use of *bacteriophages* or narrow-spectrum antibiotics) may become a future research direction, aiming to maintain gut microbiota homeostasis while reducing exposure to broad-spectrum antibiotics.

Imbalances in the gut microbiota, such as those induced by gastrointestinal infections, can increase the risk of pathogen colonization and exacerbate infections, highlighting the limitations of antibiotic-dependent therapeutic strategies. Targeted modulation of the gut microbiome through approaches including probiotics and prebiotics helps restore microbial equilibrium and competitively inhibit pathogens, thereby reducing the reliance on antibiotics and promoting a shift toward antibiotic-sparing treatment approaches (Negrut et al., 2020).

Overall, the application of SDD in the ICU needs to be combined with individualized risk assessments, weighing the relationship between reducing infectious complications and the potential risk of resistance. Future research should further clarify the clinical effects of probiotic interventions and optimize SDD protocols through rigorous monitoring of resistance evolution, in order to achieve the synergistic goals of infection control and microbial ecological protection.

### 4.3 Multimodal strategy of infection control

In the antibiotic de-escalation strategies for multidrug-resistant organism (MDRO) infection prevention, multimodal transmission-blocking approaches integrating artificial intelligence (AI)-driven hand hygiene compliance monitoring with cost-effectiveness analyses of contact isolation/patient cohorting have emerged as critical interventions. Deep learning algorithm-based AI camera systems enable real-time analysis of healthcare workers’ hand hygiene behaviors, automatically identifying lapses in compliance during critical contact moments (e.g., pre-patient contact, post-environmental exposure) and triggering immediate audiovisual feedback. A study demonstrated (Lintz, 2023) that AI feedback systems reduced hand hygiene non-compliance rates in intensive care units (ICUs) by 32%, while generating interactive data dashboards to assist infection control teams in targeting high-risk units for focused interventions. Integration with electronic health record systems further automated audit trail generation, reducing labor costs by 78% compared to manual observation and significantly enhancing data accuracy.

Cost-effectiveness analyses of contact isolation strategies reveal trade-offs between infection control efficacy and resource utilization. Single-room isolation, while highly effective in preventing cross-transmission, requires substantial infrastructure investments. However, a Markov modeling study (Lin et al., 2022) showed that for carbapenem-resistant *Enterobacterales* (CRE)-colonized patients, single-room isolation reduced hospital-acquired infection rates by 41% compared to cohorting, with an incremental cost-effectiveness ratio of US\$12,300 per quality-adjusted life year (QALY) gained—below the willingness-to-pay threshold in high-income countries. When isolation room utilization exceeded 85%, IoT-enabled dynamic zoning management systems optimized bed allocation, decreasing isolation room vacancy rates from 18% to 6% and reducing annual depreciation costs by US\$450,000 in tertiary hospitals (Ogiela et al., 2023). In resource-limited settings, cohorting patients with identical MDRO lineages in dedicated units maintained 89% contact precaution compliance while lowering operational costs by 34% compared to single-room isolation (Gussin et al., 2024). Environmental disinfection synergies further amplified these benefits—deploying ultraviolet-C (UV-C) robots in isolation units reduced surface contamination by 92% and shortened hospital stays by 2.1 days for carbapenem-resistant *Acinetobacter* baumannii infection cases (Sun et al., 2023).

## 5 Challenges and future perspectives

**FIGURE 1:**
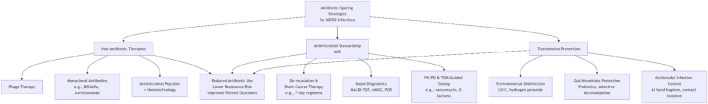
Antibiotic-sparing strategies for MDRO infections flowchart.

**FIGURE 2:**
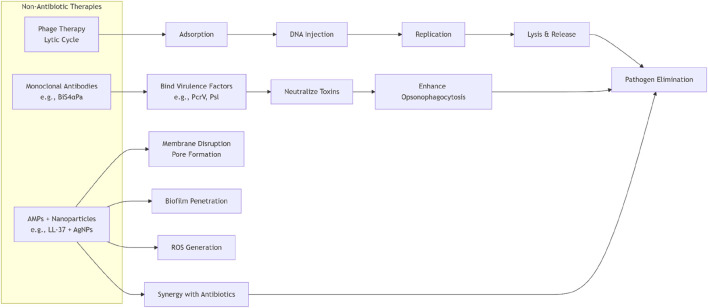
Mechanisms of non-antibiotic therapies.

As we delve deeper into the “de-antibiotic” strategies for managing infections caused by multidrug-resistant organisms (MDROs), we clearly recognize the complex challenges and broad future prospects in this field, such as Figures 1, 2. Clinical translation barriers, regulatory and ethical considerations for non-antibiotic therapies, cost-effectiveness balancing, and the exploration of combination therapies collectively constitute key issues in current research and practice.

While novel non-antibiotic therapies, such as bacteriophage therapy, demonstrate remarkable potential, their path to clinical translation is fraught with obstacles. The stringent requirements for safety, efficacy, and personalized customization of these therapies necessitate strict adherence to breakthrough therapy designation criteria set by regulatory agencies like the FDA, ensuring that patients receive the maximum benefit with minimal risk during treatment. This process not only requires robust basic scientific research as a foundation but also relies on extensive, high-quality clinical trial data to comprehensively evaluate their long-term effects and potential risks. Meanwhile, the regulatory and ethical issues surrounding non-antibiotic therapies are increasingly prominent. As a biological therapy, bacteriophage therapy involves biosafety risks in its preparation, storage, transportation, and use, as well as ethical considerations such as patients’ right to informed consent and privacy protection before receiving treatment. These are issues we must confront and resolve appropriately, requiring in-depth interdisciplinary collaboration that brings together the wisdom of microbiology, pharmacology, ethics, law, and other fields to jointly construct a scientific, reasonable, and humane regulatory framework.

In the weighing of cost-effectiveness, the construction of economic models between non-antibiotic therapies and traditional antibiotics is of particular importance. Although novel therapies like monoclonal antibodies exhibit remarkable efficacy in treating certain MDRO infections, their high research and development costs and production expenses often limit their widespread clinical application. In contrast, despite facing severe challenges of resistance, traditional antibiotics, with their mature production processes and relatively affordable prices, remain the preferred treatment option in specific contexts. Therefore, exploring more economically viable treatment regimens while ensuring efficacy has become an important task before us.

Additionally, the exploration of combination therapies has opened up new avenues for treating MDRO infections. The synergistic effects of *bacteriophages* and antibiotics offer the possibility of overcoming the limitations of monotherapies. However, the efficacy and safety of such combination therapies still require further research and practical validation. In the future, we hope to further enhance treatment effects while reducing the risk of resistance development by optimizing the combination, dosage, and administration sequence of combination therapies.

In summary, although the “de-antibiotic” strategies for MDRO infections are fraught with challenges, their future development direction is full of promise. Through interdisciplinary collaboration, technological innovation, and policy guidance, we hope to overcome the current obstacles and explore safer, more effective, and economical treatment regimens. In this process, continuous research investment, a stringent regulatory system, and extensive international cooperation will serve as the key driving forces propelling this field forward.
